# Inhibition of phenylpropanoid biosynthesis increases cell wall digestibility, protoplast isolation, and facilitates sustained cell division in American elm (*Ulmus americana*)

**DOI:** 10.1186/1471-2229-12-75

**Published:** 2012-05-30

**Authors:** A Maxwell P Jones, Abhishek Chattopadhyay, Mukund Shukla, Jerzy Zoń, Praveen K Saxena

**Affiliations:** 1 Gosling Research Institute for Plant Preservation, Department of Plant Agriculture, 50 Stone Rd East, University of Guelph, Guelph, ON, Canada, N1G 2W1; 2 Laboratory of Molecular Chemistry, Department of Medicinal Chemistry and Microbiology, Faculty of Chemistry, Wrocław University of Technology, Wybrzeze Wyspiańskiego 27, PL-50-370, Wrocław, Poland

**Keywords:** Hydroxycinnamic acid, 2-aminoindane-2-phosphonic acid, Protoplast, Cell wall, Digestibility, American elm

## Abstract

**Background:**

Protoplast technologies offer unique opportunities for fundamental research and to develop novel germplasm through somatic hybridization, organelle transfer, protoclonal variation, and direct insertion of DNA. Applying protoplast technologies to develop Dutch elm disease resistant American elms (*Ulmus americana* L.) was proposed over 30 years ago, but has not been achieved. A primary factor restricting protoplast technology to American elm is the resistance of the cell walls to enzymatic degradation and a long lag phase prior to cell wall re-synthesis and cell division.

**Results:**

This study suggests that resistance to enzymatic degradation in American elm was due to water soluble phenylpropanoids. Incubating tobacco (*Nicotiana tabacum* L.) leaf tissue, an easily digestible species, in aqueous elm extract inhibits cell wall digestion in a dose dependent manner. This can be mimicked by *p*-coumaric or ferulic acid, phenylpropanoids known to re-enforce cell walls. Culturing American elm tissue in the presence of 2-aminoindane-2-phosphonic acid (AIP; 10-150 μM), an inhibitor of phenylalanine ammonia lyase (PAL), reduced flavonoid content, decreased tissue browning, and increased isolation rates significantly from 11.8% (±3.27) in controls to 65.3% (±4.60). Protoplasts isolated from callus grown in 100 μM AIP developed cell walls by day 2, had a division rate of 28.5% (±3.59) by day 6, and proliferated into callus by day 14. Heterokaryons were successfully produced using electrofusion and fused protoplasts remained viable when embedded in agarose.

**Conclusions:**

This study describes a novel approach of modifying phenylpropanoid biosynthesis to facilitate efficient protoplast isolation which has historically been problematic for American elm. This isolation system has facilitated recovery of viable protoplasts capable of rapid cell wall re-synthesis and sustained cell division to form callus. Further, isolated protoplasts survived electrofusion and viable heterokaryons were produced. Together, these results provide the first evidence of sustained cell division, callus regeneration, and potential application of somatic cell fusion in American elm, suggesting that this source of protoplasts may be ideal for genetic manipulation of this species. The technological advance made with American elm in this study has potential implications in other woody species for fundamental and applied research which require availability of viable protoplasts.

## Background

One of the defining characteristics of the plant kingdom is the exceptional capacity of organs, tissues, and individual cells to de-differentiate and regenerate into complete plants; a phenomenon referred to as totipotency [1]. Perhaps the ultimate expression of totipotency occurs during protoplast isolation and regeneration, where cells are liberated from their cell walls and can be induced to regenerate into whole plants as reported in more than 400 plant species [2,3]. Protoplast systems offer a unique opportunity to study fundamental aspects of plant biology such as membrane physiology, cell wall metabolism and stress responses [3], as well as serving a number of practical applications including the production of interspecific hybrids between sexually incompatible species [4-7], the development of novel genetic diversity through somaclonal-protoclonal variation [8,9], and as an alternative approach to facilitate the insertion of large pieces of DNA or organelles [10,11]. While the manipulation of protoplasts has been widely achieved in many herbaceous families such as the Solanaceae, progress has been much slower in the development of this technology for woody plants.

A potentially valuable application of protoplast technologies recognized over 30 years ago was in the case of the American elm (*Ulmus americana* L.) [12]. This species was once one of the most common and iconic species of tree planted across North America until the population was decimated by the introduction of Dutch elm disease (DED) in the mid twentieth century. Today, after more than 70 years of research and classical breeding, several DED tolerant cultivars have been released [13]. However, while these trees represent a significant advance, none are considered resistant in that they do harbour the fungus and exhibit mild symptoms. Given the immense screening and breeding efforts that have occurred, it appears that the genetic resources for true DED resistance may not be present in *U. americana* and will need to be generated through modern transgenics or hybridization with resistant species of elm. Interspecific hybridization using classical approaches has been for the most part unsuccessful because of the sexual incompatibility between American elm and other elms [14]. As such, attempts at protoplast isolation and regeneration with the ultimate goal of developing DED resistant somatic hybrids through somatic fusion have been attempted by various researchers as early as 1980 [12,15-19]. However, despite the repeated attempts by various researchers there have been no successful reports of protoplast regeneration in American elm.

One of the major challenges in developing a protoplast regeneration system in American elm, as with many other woody species, is the difficulty in efficiently and reproducibly isolating protoplasts [15,16]. While this problem has been circumvented in some species by selecting juvenile tissues or embryogenic callus [3,20], this approach has not facilitated protoplast regeneration of American elm. For example, Redenbaugh et al. [15] were not able to isolate protoplasts from young American elm leaves and when using cotyledons as the source material, less than half of their 72 attempts were successful. Further, in the cotyledon preparations where protoplasts were obtained, the isolation frequency was generally below 10%, the cell division rate was low, and the protoplasts ultimately failed to regenerate. Lange and Karnosky [16] were able to isolate American elm protoplasts from cotyledons, suspension culture, and callus tissues, but required long enzymatic incubation periods and the protoplasts ultimately failed to proliferate. The authors postulated that this recalcitrance may have been a consequence of toxic effects resulting from the long exposure to the enzyme solution. Preliminary studies conducted by Dorion et al. [18,19] reported high protoplast yields from young greenhouse grown American elms using a 17 h incubation in a more active enzyme solution containing 0.2% Onozuka RS Cellulase, 0.05% Driselase, and 0.03 Pectolyase Y23. However, these reports do not provide any indication of variability or reproducibility of the protocol, and the isolated protoplasts did not display sustained cell division. A study using similar methods reported standard deviations of protoplast yields in *U. minor* were often greater than 50% of the mean [21], indicating that this approach was highly variable in elm or failed attempts were pooled in the data. Studies conducted in our lab using young American elm leaves as described by Dorion et al. [18,19] concur with the findings of Conde and Santos [21] in that protoplast yields from young (1^st^ and 2^nd^) actively growing leaves were inconsistent regardless of the enzyme solution used, and in our experience isolations often fail completely. In order to develop protoplast regeneration and hybridization systems for American elm and other difficult woody plants it is imperative that the underlying biochemical mechanism preventing reproducible enzymatic degradation of source tissue is identified and that novel approaches are developed to facilitate reliable protoplast isolation.

Some clues about the nature of this phenomenon were provided by Butt [22], who reported that thoroughly washing chopped leaf material in water prior to enzymatic digestion significantly increased protoplast yields in four woody plant species. Further, when the washed leaves were incubated in their own wash water, the tissues regain their resistance to enzymatic digestion. Together, these data suggest the cell walls are being modified by water soluble compounds that impart resistance to enzymatic degradation. Two compounds putatively identified for their role in the resilience of cell walls are *p*-coumaric and ferulic acid. These compounds are well known for their role in cell wall structure, especially in the Poaceae [23]. Specifically, in grasses they form 4,4′-dihydroxytruxillic acid and other cyclodimers in the cell wall that make them more resistant to biodegradation in ruminants. Further, the release of preformed phenylpropanoids and/or the up-regulation of the pathway, resulting in biochemical re-enforcement of the cell wall, are well established components of plant defence responses in many species, including dicots [24-26]. This phenomenon of cell wall modulation has been observed in whole plant systems upon wounding [27] or microbial infection [25], and in cell culture systems in response to a variety of elicitors [24]. In the case of protoplast isolation, it appears that these compounds are already present in the leaves and are released upon mechanical injury incurred during tissue preparation. Thus, these compounds may modify the cell walls and inhibit cell wall degradation which can severely restrict the liberation of protoplasts.

Transgenic plants that have an inhibited phenylpropanoid pathway show greater susceptibility to pathogens and have more readily digestible cell walls [26,28]. Specifically, production of tyrosine decarboxylase, an enzyme thought to facilitate cell wall re-enforcement, has been found to be inversely related to cell wall digestibility and protoplast release in canola [26]. While transgenic technologies have helped elucidate the role of phenylpropanoids in cell wall digestability, the effects are permanent and have deleterious effects on plant fitness. As such, the current study utilized a series of competitive inhibitors of PAL, the first dedicated enzyme in the phenylpropanoid pathway, to investigate the relationship between phenylpropanoid biosynthesis and cell wall digestibility. Here we provide the first evidence that by preventing phenylpropanoid biosynthesis using PAL inhibitors, it was possible to overcome the difficulties in cell wall degradation and dramatically broaden the applicability of protoplast technology in woody plants using the American elm as a model system.

## Results and discussion

Initial attempts were made to isolate protoplasts from a wide range of American elm tissues including young leaves (1^st^ and 2^nd^ position) from actively growing in vitro plants, seedlings, and greenhouse grown plants, as well as cotyledons, hypocotyls, and seedling roots. During these initial trials a number of cell wall degrading enzymes were evaluated at different concentrations and combinations, including the reportedly more active mixture used successfully in several *Ulmus* spp. by Dorion et al. [18,19] (data not shown). While protoplasts were occasionally obtained, the results were similar to what had been previously reported in that the yields were often very low [15] and the success rate was inconsistent regardless of composition of the enzyme solution. Sometimes high yields as described by Dorion et al. [18,19] were obtained, but this was not consistent even when the protocol was the same between isolation attempts and all reasonable precautions to use uniform plant material were taken. For example, a high yield of protoplasts was obtained from freshly emerged greenhouse leaves on March 30, 2011, but the isolation completely failed 5 days later on April 4, 2011 under same experimental conditions using fresh leaves from the same group of trees. The lack of reproducibility with young freshly emerged leaves and long exposure to a range of enzyme mixtures was deemed insufficiently reliable to proceed with regeneration and fusion experiments and was the impetus for this study.

Washing young in vitro American elm (*Ulmus americana*) leaf tissue with water increased protoplast yields from an average of approximately 4000/g to 34 000/g. While this was a statistically significant increase in yield, it was far below the millions per gram reported for other species [22], it did not work consistently between isolation attempts, and was not a large enough improvement to attempt culture and fusion. The relative ineffectiveness of this procedure for American elm may indicate that the interfering compounds were present in higher amounts, were more difficult to extract, and/or there were other factors inhibiting protoplast isolation in this species. This difference was likely responsible for the difficulties that have been encountered in isolating protoplasts from American elm compared to other species of elm and woody plants [15]. As such, while this simple washing technique was capable of sufficiently removing the interfering compounds from some woody plants, including *Ulmus glabra*[22], it was insufficient for the more recalcitrant American elm. This study was aimed at elucidating the underlying biochemical mechanisms and developing a systematic approach to circumvent the problem.

**Figure 1:**
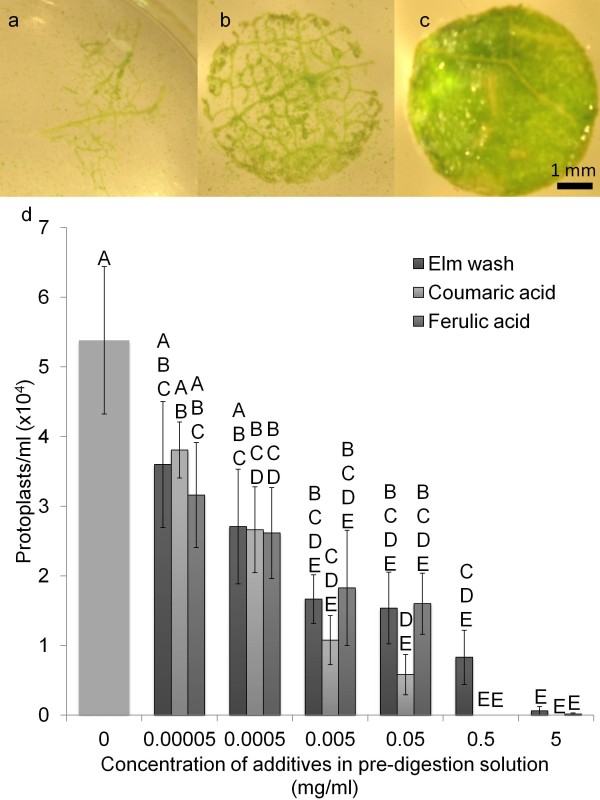
**Digestibility and protoplast isolation frequencies of tobacco leaf discs treated with elm extract or hydroxycionnamic acids.** Leaf discs were vacuum infiltrated (20 min) and incubated for 24 h in various concentrations of aqueous American elm leaf extract, *p*-coumaric acid, or ferulic acid prior to a 16 h incubation in cell wall degrading enzymes. **a**; Tobacco leaf disc incubated in sterile deionised water prior to digestion, **b**; tobacco leaf disc incubated in 0.005 mg/ml aqueous American elm extract prior to digestion, **c**; tobacco leaf disc treated with 5 mg/ml elm extract prior to digestion, **d**; Protoplast yields for tobacco leaf discs pre-incubated in various concentrations of elm extract, *p*-coumaric acid, or ferulic acid for 24 h followed by 16 h in cell wall degrading enzymes. Bars with the same letters were not significantly different based on a means separation with Tukey’s adjustment with a p-value of 0.05.

The increase in cell wall digestibility of leaves that had been leached with water can be reversed by incubating the leaf in its own extract [22]. In the current study, this phenomenon was further investigated by incubating tobacco leaf tissue, a species with a readily digestible cell wall, in an aqueous extract of American elm leaves prior to enzymatic degradation. The aqueous extract effectively inhibited cell wall digestion in a dose dependent manner (Figure 1d), supporting the hypothesis that the resistant nature of the cell wall in woody plants such as American elm was because of a water soluble chemical or group of chemicals [22]. This inhibiting effect could also be induced by using leachate from young leaves of in vitro American elm plants or leaf derived callus, indicating that this phenomenon occurs in vitro and was not limited to leaf tissues (data not shown). These observations with tobacco indicated that there was no requirement for any specialized enzymes or a fundamental difference in cell wall composition between resistant species such as American elm and a susceptible species such as tobacco. It was likely that the presence of compounds responsible for reduced digestibility would instill this trait in the cell wall of higher plants in general.

Previous evidence suggests that the resilience of the cell wall in woody plants was because of the presence of the hydroxycinnamic acids, *p*-coumaric and ferulic acid [22]. Many plants are known to release or quickly synthesize hydroxycinnamic acids in response to mechanical damage or microbial attack [25,27,29]. These compounds serve as precursors for the formation of hydroxycinnamoyl-CoAs which contribute to cell wall strengthening and lignification, ultimately reducing cell wall digestibility and inhibiting microbial infection [24,25]. Plant tissues are likely to accumulate these compounds throughout their existence as they are continuously exposed to various stresses, which may contribute to the observation that leaves become increasingly resistant to enzymatic degradation with age [18,19,21,22]. Protoplast isolation typically depends on mechanically wounding the tissue followed by incubation in cell wall degrading enzymes purified from fungi and it was likely that this process elicits the further release or synthesis of hydroxycinnamic acids which then modify the cell wall in some species.

The addition of either *p*-coumaric or ferulic acid to washed leaf tissue from woody species has been shown to re-instate resistance to cell wall digestion similar to when incubated in their own leachate [22]. Similar observations were made in the current study with tobacco where leaf tissue incubated in either compound reduced protoplast isolation in a dose dependant manner similar to American elm leaf extract (Figure 1a-d). These data support previous studies that suggest hydroxycinnamic acids were involved in re-enforcing the cell walls and increasing resistance to enzymatic degradation. This study also indicates that *p*-coumaric and ferulic acid alone were capable of increasing cell wall resilience in a species that was typically easily digested, again suggesting that incorporation into the cell wall was not dependent upon any unique characteristics of woody plants.

An alternative approach to evaluate the role of hydroxycinnamic acids in cell wall digestibility and develop an efficient approach to isolate protoplasts from American elm was to inhibit their biosynthesis. Hydroxycinnamic acids are lignin precursors produced through the phenylpropanoid pathway. While cultural factors such as light exposure, temperature, plant nutrition, and ontological development are known to influence this pathway, numerous factors were evaluated in the current study and were insufficient to facilitate a reproducible protoplast isolation protocol from American elm (data not shown). A number of competitive PAL inhibitors, namely 2-aminoindane-2-phosphonic acid (AIP [30,31]), (*S*)-2-aminooxy-3-phenylpropionic acid (AOPP, notation (*S*) and L are equal [32]) and *O*-benzylhydroxylamine (OBHA [33]) have been shown to significantly reduce the production of phenylpropanoids in a variety of species. In a previous study with *Lycopersicon esculentum* suspension cultures, the addition of AIP to the medium effectively reduced the cells ability to accumulate wall bound phenolics when challenged with a fungal elicitor [34]. As such, these three inhibitors were included in the growth medium to assess their influence on cell wall digestibility and protoplast isolation frequency in American elm suspension cultures.

**Figure 2:**
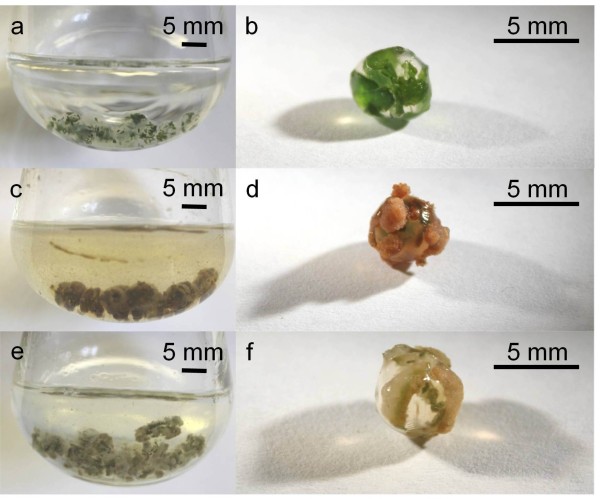
**Two-phase suspension culture of American elm (*Ulmus americana*) with and without AIP.** Cultures were started with leaf tissue embedded in alginate beads and cultured in liquid MSO media supplemented with 5 μM BA, 1 μM NAA: **a**; Freshly prepared beads in flask, **b**; close-up of freshly prepared bead, **c**; suspension culture developed from beads, **d**; close-up of bead showing callus development, **e**; suspension culture developed in medium supplemented with 150 μM AIP, **f**; close-up of bead grown in 150 μM AIP showing callus development.

**Figure 3:**
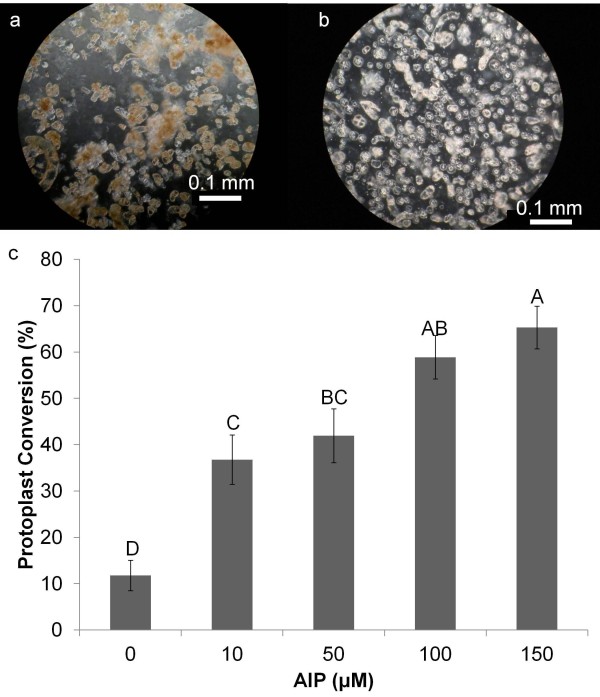
**Protoplast isolation rates of American elm callus grown in various concentrations of AIP.** American elm callus cells were grown on MSO basal medium supplemented with 5 μM BA, 1 μM NAA, and various levels of AIP and the percentage that developed into protoplasts after a 4 h incubation in a cell wall degrading enzyme mixture were counted. **a**; American elm callus grown without AIP after digestion, **b**; American elm callus grown on 100 μM AIP after digestion, **c**; Protoplast conversion rates for American elm callus grown on varying levels of AIP. Bars with the same letters were not significantly different based on a means separation with Tukey’s adjustment with a p-value of 0.05.

Initial studies indicated that all three PAL inhibitors were deleterious to the growth of in vitro elm plants, and insufficient leaf tissue was produced for further experiments. Similar observations have been made for birch (*Betula pubescens*) seedlings where growth was almost completely inhibited in the presence of 30 μM AIP [35]. Consequently, further experiments were conducted using a two phase suspension culture system in which leaf tissue was embedded in alginate beads suspended in liquid culture medium (Figure 2a-f) to produce the source callus tissue. Of the three inhibitors, AIP was the most effective and facilitated cell wall digestion and protoplast isolation from American elm callus in a dose dependant manner, increasing the digestion rate from 11.8% (±3.27) in the controls to 65.3% (±4.60) in callus grown in 150 μM AIP (Figure 3c). Addition of AOPP resulted in a modest increase in protoplast isolation while OBHA had no beneficial effect (data not shown). These data support previous studies that found AIP to be more effective at inhibiting PAL than the other two compounds [31].

**Figure 4:**
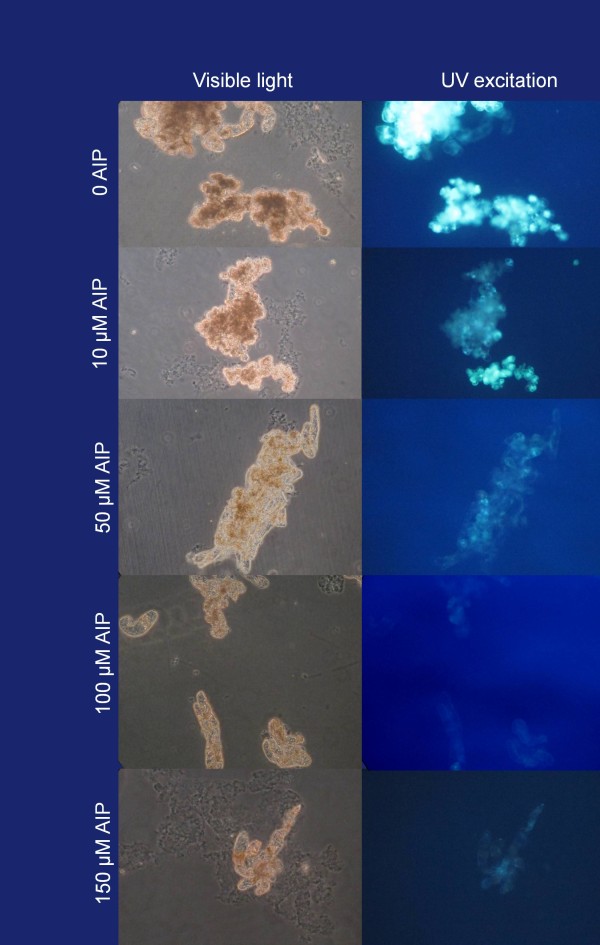
**Visualization of flavonoids in American elm callus.** American elm callus was grown in liquid MSO basal medium supplemented with 5 μM BA, 1 μM NAA, and various levels of AIP. The callus was then stained with natural product reagent (NPR) and viewed under visible light with and without UV excitation. Tissue stained with NPR fluorescing yellow under UV excitation indicates the presence of flavanoids.

The increase in cell wall digestibility with increased levels of AIP was accompanied by a reduction in the flavonoid content in the tissue when stained with NPR (Figure 4). While the flavonoid content in and of its self was unlikely to influence cell wall digestibility, flavonoids are also synthesized through the phenylpropanoid pathway and have been used as an indicator for the activity of the pathway [36]. While total phenol content had been used in this capacity in previous reports, AIP was found to interfere with this assay at the concentrations used in this study (data not shown). The callus produced in the presence of AIP also remained creamy white in colour (Figure 2e-f). This was in stark contrast to the brown coloration observed in the control callus (Figure 2c-d), indicating that AIP was inhibiting the accumulation of polyphenols. Together, the inverse relationship between both flavonoid and polyphenol contents with protoplast isolation rates strongly suggests that the factor inhibiting enzymatic digestion of the cell wall in American elm was a product of the phenylpropoanoid pathway. Cell wall digestibility was significantly increased by selectively inhibiting this pathway which facilitated the development of an efficient, reproducible protocol for the isolation of American elm protoplasts.

**Figure 5:**
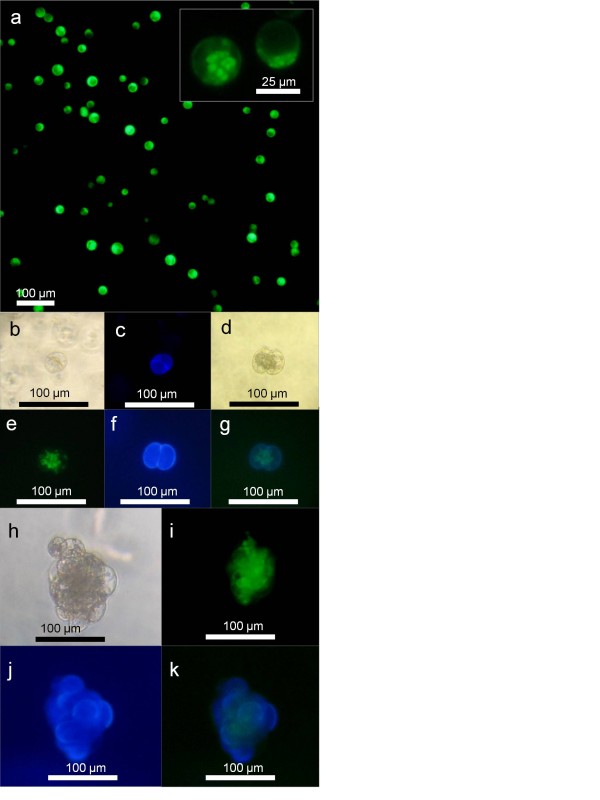
**Protoplasts and developing cells obtained from American elm callus.** Images depict American elm tissue cultured in MSO basal medium supplemented with 5 μM BA, 1 μM NAA, and 100 μM AIP after a 4 h digestion in cell wall degrading enzymes. **a**; Initial purified protoplast preparation stained with FDA for viability, cells fluorescing green indicate viability, **b**; Individual cell after 2 days of culture under visible light, **c**; Same cell as depicted in ‘b’ stained for cellulose with calcofluor white, blue fluorescence indicates cell wall formation, d; Protoplast that has completed one cell division 6 days after culture initiation, **e**; Cells depicted in ‘d’ stained with FDA for viability, **f**; Cells depicted in ‘d’ stained for cellulose with calcofluor white, **g**; cells depicted in ‘d’ stained for viability with FDA and cellulose with calcofluor white. **h**; Protoplast derived callus 14 days after culture initiation, **i**; Callus depicted in ‘h’ stained with FDA for viability, **j;** Callus depicted in h stained for cellulose with calcofluor white, **k**; Callus depicted in ‘h’ stained for viability with FDA and cellulose with calcofluor white.

While the approach of using AIP has dramatically increased our ability to consistently obtain large numbers of protoplasts from American elm, inhibiting the phenylpropanoid pathway with AIP is known to reduce the accumulation of biomass in a number of species [35,36]. In order for this technology to have practical application in developing protoplast regeneration systems in difficult woody species, it was critical to examine the viability and growth potential of the resulting protoplasts. In the current study, protoplasts obtained from tissue cultured in the presence of 100 μM AIP had a relatively high viability, typically ranging from about 80% to over 90% based on fluorescein diacetate (FDA) staining (Figure 5a). The viability observed in the protoplasts derived from this system was comparable to the upper levels observed in other species of *Ulmus* where protoplast regeneration has been successful [18,21].

This relatively high viability may be a consequence of obtaining protoplasts from young actively growing callus tissue, the reduced duration of exposure to the potentially deleterious cell wall degrading enzyme solution, or a combination of these and other factors. Whereas most protoplast isolation protocols in American elm require 4 to 48 h of incubation in enzyme solution [12,15-19], in our system this can be reduced to 1-4 h. While successful protoplast isolation from American elm was generally inconsistent and often worked less than half of the time in previous attempts [15], the suspension cultures grown in the presence of AIP have reliably yielded viable protoplasts on a bi-weekly basis for more than 5 months and continue to be productive.

Protoplasts isolated using this system and suspended in low melting point agarose beads cultured in liquid Kao and Michayluk [37] medium supplemented with 5 μM NAA and 5 μM BA started to re-develop cell walls within 2 days and showed early signs of cell division (Figure 5b,c). Efficient re-synthesis of the cell wall is a pre-requisite for cytokinesis in protoplasts and is influenced by the conditions used for cell wall digestion and isolation [38]. Previous studies have reported that cell wall formation in American elm protoplasts occurs sporadically and starts later, between 4 and 21 days post-culture [15]. After 6 days of culture in the current system, 28.5% (±3.59) of the protoplasts had initiated cell division and well developed cell walls were present (Figure 5d-g). This compares favourably to previous studies where American elm protoplasts had much lower division rates, as low as 1%, was only observed in some preparations, and started 9-21 days after initial culture [15]. In previous studies where cell division was observed, the cells failed to continue to divide and there has been no previous report of protoplast-derived callus regeneration in this species [15,16,18,19]. In the current system, the cells continued to divide and protoplast-derived calli were produced by day 14 (Figure 5h-k). As such, this protocol represents the first report of callus regeneration from American elm protoplasts more than 30 years after the first attempt.

**Figure 6:**
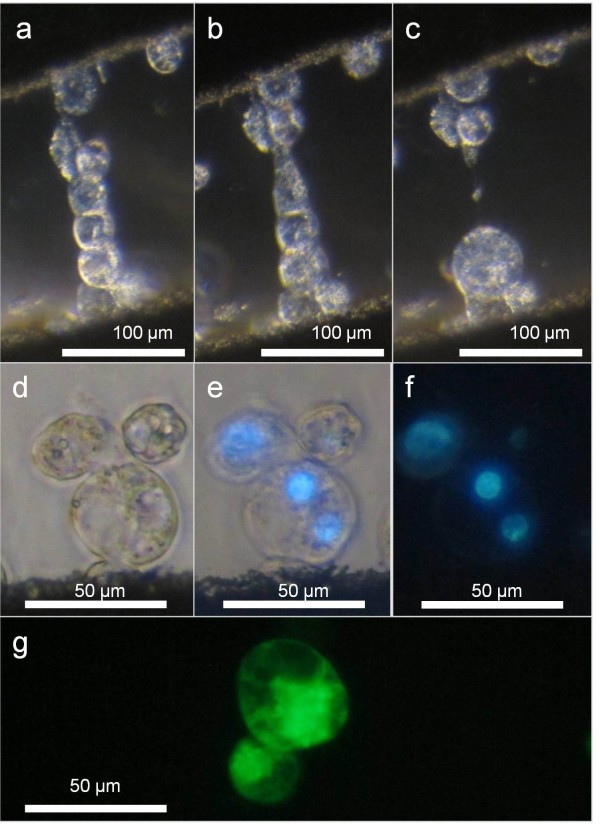
**Electrofusion of American elm protoplasts.** American elm protoplasts, **a**; aligned in AC current, **b**; shortly after DC pulses, **c**; fused together after DC pulses, **d-f**; fused heterokaryon with nuclei stained with DAPI with UV excitation and decreasing light levels, and **g**; putitive fusion product embedded in agarose bead stained with FDA for viability.

The consistent supply of protoplasts has also facilitated initial studies into protoplast fusion technologies to realize the long term-goal of producing interspecific elm hybrids which may exhibit resistance to DED [12,15-19]. Thus far, we have optimized various electrofusion parameters to conduct fusion experiments with American elm protoplasts. The process of somatic fusion using electroporation can be detrimental to protoplasts, particularly those that are less viable and unable to withstand the repeated centrifugation and culture manipulations required. Stable heterokaryons have been observed (Figure 6c-g), putative hybrid cells remained viable after being transferred into agarose beads (Figure 6g), and initial signs of cell division have been observed in protoplasts that have been exposed to the electrofusion procedure. While electrofusion parameters need further optimization to maximize fusion while minimizing cell lysis, these preliminary results indicate that protoplasts produced through this system were sufficiently robust to survive electrofusion and will lay the foundation to initiate somatic fusion with DED-resistant species of elm.

It was difficult to make direct comparisons between this study and previous efforts to regenerate *U. americana* protoplasts because of different source material, media, plating densities, and culture systems. Nevertheless, sustained proliferation of protoplasts (derived through this protocol) into calli compared to previous unsuccessful attempts may be a result of the reduction of polyphenols in the source tissue, which are known to inhibit subsequent protoplast division [39]. As well, a shorter period of incubation with much less stress on the protoplasts may contribute to their higher growth response. Regardless of the mechanisms, the reproducibility of the current system in providing regenerable protoplasts represents a significant step forward and a solid foundation to develop a protoplast manipulation system for American elm. This development could ultimately facilitate the development of DED resistant somatic hybrids, cybrids, and provide an alternate avenue to insert large segments of DNA. Protoplast based systems have been used to generate novel germplasm with disease or insect resistance in a range of species such as potato [40], tobacco [41], *Brassica* spp. [42], and citrus [7]. Given the multigenic response involved in DED resistance, together with the long life span of *U. americana* relative to the rapidly evolving pathogen, protoplast technologies are particularly appealing to integrate stable disease resistance traits found in some Asiatic *Ulmus* species into *U. americana*[43,44].

## Conclusions

The significance of the current study lies in its innovative and systematic approach to develop an effective solution to a problem which has limited the progress in protoplast based genetic improvement of an important plant species for many decades. The data presented here emphasize the critical role of the phenylpropanoid pathway in modifying cell walls in American elm in such a way that inhibits enzymatic degradation and has slowed progress toward the development of protoplast culture and fusion in this species despite repeated attempts for over 30 years. The crucial advancement made in this study was selective inhibition of the phenylpropanoid pathway by AIP, a potent phenylalanine ammonia lyase inhibitor, thereby facilitating cell wall degradation and subsequent release of protoplasts from callus tissue in large numbers and a very short period of enzymatic incubation. Protoplasts isolated using this system displayed high rates of viability, initiate cell division sooner and at much higher frequencies than reported earlier, and have facilitated the first report of protoplast-derived callus in this species. This technological advance has enhanced our ongoing research to develop protoplast regeneration and fusion systems for the eventual development of DED-resistant somatic hybrids. The fundamental aspect of this technology also provides a novel approach to expand the application of inhibitors of phenylpropanoid pathway to many traditionally recalcitrant woody species in which cell wall digestion and reproducible protoplast isolation has proven to be very difficult, if not impossible. Ongoing studies indicate that this approach increases protoplast isolation in other woody species including sugar maple (*Acer saccharum*) and hazelnut (*Corylus* sp.).

## Methods

### Plant stock material

*Ulmus americana* (American elm) and *Nicotiana tabacum* (tobacco) tissues were obtained from plants in an in vitro germplasm collection maintained at the University of Guelph (Guelph, ON, Canada). The *U. americana* used in the study included a variety of accessions maintained on DKW [45] medium (D190; *Phyto*Technology Laboratories, Lenexa, KS, USA) supplemented with 3% sucrose, 2.2 μM BA (Sigma-Aldrich, Canada), and 0.3 μM GA_3_ (Sigma-Aldrich, Canada) as previously described [46]. For *U. americana* seedling studies, seeds collected from a mature tree growing on the University of Guelph campus were surface disinfested in 10% commercial bleach (5.5% sodium hypochloride) followed by three rinses in sterile distilled water before being cultured in GA-7 vessels (Magenta Chicago, IL, USA) containing 40 ml of basal MSO [47] medium with 3% sucrose. All *N. tabacum* plants used in this study were accession PetH4 and were maintained on basal MSO [47] medium (M519; *Phyto*Technology Laboratories, Lenexa, KS, USA) with 3% sucrose. All above media were solidified with 2.2 g/l phytagel (SigmaAldrich, Canada) adjusted to a pH of 5.7 prior to being autoclaved at 121°C and 21 psi for 20 min. The cultures were maintained in a growth room at 24°C ± 2°C under a 16 h photoperiod (40 μmol m^2^s^−1^) provided by cool-white fluorescent lamps (Philips Canada, Scarborough, ON).

### Elm leaf wash and digestion

In initial digestion studies, young leaves (1^st^ and 2^nd^) of actively growing in vitro and greenhouse grown *U. americana* plants were used to evaluate the effect of thoroughly washing with water on cell wall digestibility. Greenhouse leaves were first surface disinfested in 10% commercial bleach (5.5% sodium hypocholoride) for 5 min, followed by three rinses in sterile distilled water, while in vitro leaves were used without surface disinfestation. The leaves were finely chopped in a small amount of sterile distilled water, weighed and transferred to a Petri dish (100 mm X 15 mm; Fisher Scientific, Canada) containing 20 ml of sterile water. The Petri dishes were then placed on a rotary shaker at 100 rpm for 1.5 h, during which the water was replaced with an equal volume every 30 min. The water was then removed and the tissue was weighed before being transferred into 12 ml cell wall degrading enzyme solution in a 100 mm Petri dish. In initial attempts the isolation of protoplasts was carried out using an enzyme solution comprised of cell and protoplast washing (CPW) salts [48], 91 g/l mannitol (Sigma-Aldrich, Canada), 50 mg/l 2-(*N*-morpholino) ethanesulfonic acid (MES) buffer (Sigma-Aldrich, Canada), 10 g/l Cellulase Onozuka R-10 (*Phyto*Technology Laboratories, Lenexa, KS, USA), 1.34 g/l Macerozyme R-10 (*Phyto*Technology Laboratories, Lenexa, KS, USA), and 5 g/l Driselase (Sigma-Aldrich, Canada). The enzyme solution was adjusted to pH 5.5 and filter-sterilized using a 0.22 μm vacuum filtration system (Whatman Klari-Flex, Fisher Scientific, Canada) prior to use. After initial failed isolation attempts the enzyme solution was changed to include 1%, then 2% of each of the following enzymes: Driselase (Sigma-Aldrich, Canada), Cellulase Onozuka R-10, Cellulysin^(R)^ (Calbiochem), Macerozyme R-10, Viscozyme (Sigma-Aldrich, Canada), and Macerase^TM^ (Calbiochem). Additionally, the reportedly more effective enzyme mixture described by Dorion et al. [18] was used in several attempts to digest leaves from greenhouse and in vitro leaves. The tissue was incubated in the enzyme solutions in the dark on an orbital shaker at 10 rpm (Belly Dancer, Stovall Life Science Inc., Greensboro, NC, USA) for 18 h. At this time the protoplasts were counted using a hemocytometer (Bright-Line, Horsham, PA, USA) on a compound light microscope (Photomicroscope III, Carl Zeiss Canada Ltd., Toronto, ON, Canada) and used to calculate the number of protoplasts isolated per gram of leaf tissue.

### Elm leaf wash preparation for tobacco leaf disc digestion

The elm leaf wash used to incubate tobacco leaf discs was prepared from a composite sample of leaves from 1-2-year-old trees growing in the greenhouse at the University of Guelph, Guelph, ON, Canada. The sample, weighing 8.9 g, included a range of young freshly emerged to older fully expanded leaves. The leaves were chopped into fine pieces in 400 ml distilled water using a commercial blender (model 33BL73 (7011 C), Waring, Torrington, CT, USA). The water from the blender was passed through a Buchner funnel to remove the tissue and collected in a filter flask. The tissue was washed with another 50 ml of water, transferred into 200 ml of water in a beaker, and agitated for 1 h using a magnetic stir bar. This process was repeated two more times for 1 h and then 30 min. All of the water extracts were combined and filtered through a glassfibre prefilter (Sartorius, Goettingen, Germany) followed by a 0.22 μm vacuum filtration system to remove leaf debris. The aqueous extract was then frozen, lyophilized, and stored at −80°C. The extracts were re-suspended in distilled water at desired concentrations and sterilized using a syringe filter system (0.22 μm, Fisher Scientific, Canada) before use.

### Tobacco leaf disc incubation and digestion

Tobacco leaf discs with a diameter of 5 mm were taken from fully expanded leaves using a core borer with care taken to avoid the midrib. The leaf discs were transferred abaxial side down into 0.5 ml of sterile distilled water or aqueous solutions of *p*-coumaric acid, ferulic acid, or elm leaf wash at concentrations of 0.00005, 0.0005, 0.005, 0.05, 0.5, or 5 mg/ml in 6-well culture plates (Corning Inc., Corning, NY, USA). The plates were placed in a vacuum desiccator and vacuum infiltrated for 20 min, followed by a 24 h incubation in the dark at room temperature. After the 24 h incubation period, the leaf discs were transferred abaxial side down into 24-well culture plates (Corning Inc., Corning, NY, USA) containing 0.5 ml/well of enzyme solution comprised of CPW salts [47], 91 g/l mannitol, 500 mg/l MES buffer, 10 g/l Cellulase Onozuka R-10, 1.34 g/l Macerozyme R-10, and 5 g/l Driselase,adjusted to pH 5.5, and filter-sterilized. The leaf discs were then incubated in the dark for 16 h, at which time the protoplasts released were quantified using a hemocytometer on a compound light microscope.

### American elm suspension culture

Callus cultures of American elm were initiated using a two-phase suspension culture system. Leaf material was blended into fine pieces of tissue in sterile water using a commercial blender for approximately 5 s. The slurry was filtered through an autoclaved Buchner funnel covered with 100 μm nylon mesh to remove the aqueous portion, and the remaining tissue was rinsed with sterile distilled water. The macerated leaf tissue was then re-suspended in sterile distilled water and added to a sodium alginate solution (48 g/l sodium alginate (Acros Organics, Belgium), 700 mg/l MES buffer adjusted to pH 5.7) at a ratio of 1:1, homogenized, and transferred drop wise into a solution containing 10 g/l CaCl_2_.2H_2_O (*Phyto*Technology Laboratories, Lenexa, KS, USA) and 700 mg/l MES buffer adjusted to pH 5.7. The alginate-leaf mixture was left in the CaCl_2_ solution for 20 min resulting in solidified alginate beads with leaf tissue embedded (Figure 2a,b). The CaCl_2_ solution was then removed and the beads were rinsed twice with sterile distilled water. Twenty alginate beads were added to 125 ml Erlenmeyer flasks each containing 20 ml of MSO media supplemented with 5 μM BA, 1 μM NAA (Sigma-Aldrich, Canada), and 0, 10, 50, 100, or 150 μM 2-aminoindane-2-phosphonic acid (AIP), L-2-aminooxy-3-phenylpropionic acid (AOPP), or *O*-benzylhydroxylamine hydrochloride (OBHA) added prior to autoclaving. The AIP was synthesized as described earlier [29,49], while AOPP and OBHA were purchased from Wako Pure Chemical Industries, Ltd., Osaka, Japan and Sigma-Aldrich, Canada, respectively. All cultures were maintained in the dark on a rotary shaker set at 100 rpm and all media were adjusted to pH 5.7 prior to being autoclaved for 20 min at 121°C and 21 psi. Each treatment was replicated four times and suspension cultures developed in approximately 3 weeks before being used for digestion studies.

### Digestion of American elm suspension cultures

American elm suspension cultures were transferred from the Erlenmeyer flasks into 50 ml centrifuge tubes (Fisher Scientific, Canada) and pelleted by centrifugation for 6 min at 2500 rpm (HN-SII, IEC, USA). The growth medium was then decanted and replaced with an enzyme solution [18], comprised of CPW salts [48], 91 g/l mannitol, 500 mg/l MES buffer, 2 g/l Cellulase Onozuka RS (*Phyto*Technology Laboratories, Lenexa, KS, USA), 1 g/l Driselase, and 0.3 g/l Pectolyase Y-23 (*Phyto*Technology Laboratories, Lenexa, KS, USA) adjusted to pH 5.5 and filter-sterilized. In initial studies, the tissue was incubated for 16 h, but early observations indicated that there was sufficient digestion after 4 h. As such, to reduce the potentially deleterious effects of long incubation periods, the current study utilized a 4 h incubation period. To quantify the digestion rate, two 100 cell counts were taken for each digestion using an inverted epi-fluorescent microscope (Axiovert 200, Carl Zeiss Canada Ltd., Toronto, ON, Canada), counting the percentage of cells that had converted into protoplasts.

### Protoplast isolation and purification of American elm cell suspension culture

For protoplast isolation and purification studies, American elm cell suspension cultures grown in the presence of 100 μM AIP were used. The suspensions were transferred from the Erlenmeyer flasks into 50 ml centrifuge tubes and the cells pelleted by centrifugation for 6 min at 2500 rpm. The growth medium was decanted and replaced with the enzyme mixture used for American elm digestion studies described previously. After 2 h in the enzyme solution maintained in the dark, the solution was passed through a 100 μm nylon filter into 50 ml centrifuge tubes (Fisher Scientific, Canada). The filtered suspension was pelleted by centrifugation for 6 min at 900 rpm. The enzyme solution was removed using a pipette (Fisher Scientific, Canada) and the pellet was gently transferred along with residual enzyme solution to create a layer on top of 35 ml of CPW solution [48] supplemented with 210 g/l sucrose in a 50 ml centrifuge tube. The tube was centrifuged at 900 rpm for 10 min to isolate the protoplasts by differential density gradient centrifugation. The band that formed at the interface of the two media was carefully collected using a 1000 μl pipette equipped with a wide mouth tip (Ultident Scientific, St. Laurent QC, Canada). The collected protoplasts were transferred into a 15 ml centrifuge tube (Fisher Scientific, Canada) which was then topped up to 10 ml with CPW [48] solution containing 100 g/l mannitol. The solution was mixed and the protoplasts pelleted as described previously. This washing step was repeated to remove any residual enzyme, and the pellet was reconstituted in 1 ml CPW. The cell density was determined from a subsample using a hemocytometer on a compound light microscope. Another subsample was stained for viability by adding 60 μl/ml of 2 mg/ml fluorescein diacetate (FDA; Sigma-Aldrich, Canada) dissolved in acetone (Sigma-Aldrich, Canada) and incubating the sample in the dark for approximately 10 min. Viability was determined using a 100 cell count on an inverted epi-fluorescence microscope using either a Yellow/GFP/BP or a Fitc/Bodipy/Fluo 3/Dio filter set (Chroma, Bellows Falls, VT).

### American elm protoplast culture

Protoplasts were adjusted to a density of 4 x 10^5^ cells per ml in CPW [47] solution containing 100 g/l mannitol. This was mixed with a CPW solution containing 1.6% low melting temperature SeaPlaque^(R)^ agarose (Mandel Scientific, Guelph ON, Canada) maintained at a temperature of 38Â°C to reach a final cell density of 2 x 10^5^ and concentration of 0.8% agarose. This mixture was mixed well using a wide mouth pipette and transferred drop wise to 24-well culture plates with one drop (approximately 100 μl/drop) in each well. To each well, 0.5 ml of filter sterilized KM [37] medium containing 100 g/l mannitol, 5 μM BA, and 5 μM NAA and adjusted to pH 5.7 was added after the agarose had solidified for approximately 20 min. The cultures were maintained in the dark on an orbital shaker at 10 rpm (Belly Dancer, Stovall Life Science Inc., Greensboro, NC, USA) at a temperature of 24°C. Each day, 30 μl of 2 mg/ml FDA and 0.1 mg/ml calcoflour white (Sigma-Aldrich, Canada) solutions were added to a single well and incubated in the dark for approximately 10 min. The stained cells were observed for cell viability as described previously, and for cell wall deposition by viewing on an inverted epi-fluorescence microscope using a DAPI/Hoescht/AMCA filter set (Chroma, Bellows Falls, VT). Further, the stained cells were viewed using a wide UV excitation with longpass emission filter set (Chroma, Bellows Falls, VT) to simultaneously view viability and cell wall development. At day 6 of culture, the percentage of protoplasts that had undergone division was quantified using a 100 cell count for each well. On day 9 the culture medium was replaced with fresh medium of the same composition with the exception that it contained only 5% mannitol. On day 14 colonies were again stained and viewed as described previously and the medium was replaced with fresh medium with no mannitol added.

### Electrofusion parameters

Electrofusion experiments were conducted using an Electro Cell Manipulator 2001 (BTX Harvard Apparatus, Holliston, MA). Isolated protoplasts were pelleted by centrifugation for 6 min at 900 rpm and re-suspended in a 0.6 M solution of mannitol at a cell density of 5 x 10^5^cells/ml. In initial experiments 20 μl aliquots of the solution were transferred onto 0.2 mm gap size meander fusion chambers (BTX Harvard Apparatus, Holliston, MA) that had been sterilized for 30 min in 95% ethanol and air dried. In some cases (Figure 6 d-f), the cell nuclei were stained with 4′,6-diamidino-2-phenylindole (DAPI) to verify cell fusion. A variety of parameters were adjusted over repeated fusion attempts, and the following conditions were used to produce Figure 6a–f: two cycles of 30 s 3 V AC current to align cells, followed by two 25 μs pulses of 30 V DC, then 5 s of 3 V AC. In larger scale fusion experiments (Figure 1g), 700 μl of the protoplast suspension was transferred to a 3.2 mm gap size fusion chamber (BTX Harvard Apparatus, Holliston, MA) that had been sterilized for 30 m in 95% ethanol and air dried. The parameters used in the smaller plates were scaled up to compensate for the larger gap size and the following cycle was repeated twice: 30 s of 38 V AC to align cells, two 25 μs pulses of 480 V DC, and 5 s of 38 V AC. Cells from the bulk fusion were left in the chamber for 1 h post-fusion before being gently transferred into KM medium [37], pelleted by centrifugation for 6 min at 900 rpm, and cultured as described previously for protoplasts.

### Visualization of flavonoids

A 1 ml aliquot of 2% natural product reagent (NPR; aminoethyl diphenylborinate) dissolved in 95% ethanol (Commercial Alcohols, Brampton ON, Canada) was added to 1 ml of American elm suspension cultures grown on various levels of AIP and mixed. The cells were incubated in the dark for approximately 10 min and then viewed on an inverted epi-fluorescence microscope using a wide UV excitation with longpass emission filter set (Chroma, Bellows Falls, VT).

### Experimental design and statistical analysis

Experiments conducted in 6- well or 24-well plates were arranged in a complete randomized block design with four blocks. Experiments conducted in flasks were arranged in a completely randomized design with four replicates for each treatment. All experiments were conducted at least twice, and after observing similar trends among replicate experiments the data were pooled prior to analysis. Statistical analyses were conducted using JMP 9.0.2 (SAS Institute Inc., Cary, NC). Following an ANOVA, means separations were conducted using Tukey’s adjustment with a p-value of 0.05.

## Authors’ contributions

AJ conceived the study, oversaw and developed experimental procedures, conducted statistical analyses, and drafted the manuscript. AC contributed to conceptualization of the study, conducted many of the experiments, refined procedures, and helped draft the manuscript. MS helped troubleshoot experimental procedures, supplied plant tissues, and participated in drafting the manuscript. JZ synthesized the AIP, provided support on experimental setup, and participated in drafting the manuscript. PS oversaw the project, aided in conceptualization of the project, and participated in drafting the manuscript. All authors have read and approved the final manuscript.
